# HTLV-1 Tax Oncoprotein Inhibits the Estrogen-Induced-ER α-Mediated BRCA1 Expression by Interaction with CBP/p300 Cofactors

**DOI:** 10.1371/journal.pone.0089390

**Published:** 2014-02-21

**Authors:** Meital Shukrun, Azhar Jabareen, Ammar Abou-Kandil, Rachel Chamias, Mordechai Aboud, Mahmoud Huleihel

**Affiliations:** Shraga Segal Department of Microbiology and Immunology, Faculty of Health Sciences, Ben Gurion University of the Negev, Beer Sheva, Israel; University of Dundee, United Kingdom

## Abstract

BRCA1 is a multifunctional tumor suppressor, whose expression is activated by the estrogen (E2)-liganded ERα receptor and regulated by certain recruited transcriptional co-activators. Interference with BRCA1 expression and/or functions leads to high risk of breast or/and ovarian cancer. Another multifunctional protein, HTLV-1Tax oncoprotein, is widely regarded as crucial for developing adult T-cell leukemia and other clinical disorders. Tax profile reveals that it can antagonize BRCA1 expression and/or functionality. Therefore, we hypothesize that Tax expression in breast cells can sensitize them to malignant transformation by environmental carcinogens. Here we examined Tax effect on BRCA1 expression by testing its influence on E2-induced expression of BRCA1 promoter-driven luciferase reporter (BRCA1-Luc). We found that E2 strongly stimulated this reporter expression by liganding to ERα, which consequently associated with BRCA1 promoter, while ERα concomitantly recruited CBP/p300 to this complex for co-operative enhancement of BRCA1 expression. Introducing Tax into these cells strongly blocked this E2-ERα-mediated activation of BRCA1 expression. We noted, also, that Tax exerted this inhibition by binding to CBP/p300 without releasing them from their complex with ERα. Chip assay revealed that the binding of Tax to the CBP/p300**-**ERα complex, prevented its link to AP1 site. Interestingly, we noted that elevating the intracellular pool of CBP or p300 to excessive levels dramatically reduced the Tax-mediated inhibition of BRCA1 expression. Exploring the mechanism of this reduction revealed that the excessive co-factors were sufficient to bind separately the free Tax molecules, thus lowering their amount in the CBP/p300**-**ERα complex and relieving, thereby, the inhibition of BRCA1 expression.

## Introduction

Breast cancer is a complex malignancy with several phenotypic characteristics that might be determined by several gene products and certain additional intrinsic or/and external factors [1]. However, malfunction of the first discovered breast cancer sensitivity gene product, BRCA1, has been recognized as the most frequent risk factor for this mammary tumorigenesis. Between 5 to 10% of breast cancer cases arise at early stages of women’s life in hereditary manner due to inheriting germ-line genetic factors. Approximately half of these early breast cancers proved to emerge from germ-line mutations within the BRCA1 gene, which hamper the expression or functions of its protein [2]. The remaining cases emerge sporadically at old ages with incidence being affected by various risk factors, like diet, alcohol consumption, tobacco smoking, number of pregnancies, duration of breast-feeding, predisposition to environmental and occupational pollutions and certain others [3]. Of note, however, although mutations within the BRCA1 gene are rare (2–3%) in such sporadic cases [2], the level of BRCA1 protein in cancerous breast cells of 30–40% of the sporadic breast cancers is markedly reduced by various non-mutational down-regulating mechanisms [4], indicating that BRCA1 malfunction is a major risk factor which is associated with sporadic breast cancers as well.

BRCA1 is a multifunctional protein that is involved in many cellular processes, such as gene expression [5], ubiquitination [6], host genome stabilization by enhancing DNA repair [7], securing proper centrosome amplification [8]and mitotic spindle checkpoint [8], enhancing stress-induced cell cycle arrest [9] and apoptosis [10]. With these multiple functions that protect such vitally crucial cellular processes from potentially cancer-inducing factors, BRCA1 serves as a tumor suppressor whose functional loss by germ-line mutations or sporadic down-regulating mechanisms, renders its ovarian or mammary host cells susceptible to malignant transformation. Notably, however, although BRCA1 is ubiquitously expressed in almost all tissues of the two genders, its tumor-suppressor functions are paradoxically oriented almost exclusively towards the female mammary and genital organs. The bio-molecular basis of this intriguing tissue and gender specificity is still under intensive investigation [11].

Estrogen (E2) activates the E2 receptor alpha (ERα) by liganding to it. Such activated ERα is a potent transcription factor that can activate a wide range of E2-responding genes in two alternative pathways; a classical and non-classical. The classical pathway starts by direct binding of the activated ERα to the DNA at E2-responsive elements (EREs) residing in the target promoters, which is, then, followed by recruitment of appropriate co-activators and co-factors which cooperatively stimulate the transcription of the respective gene [12]. In the non-classical pathways, the E2-liganded ERα can indirectly associate with a range of alternative non-ERE elements through interacting with their bound specific transcription factors and recruiting various co-activators and co-factors, which enhance the activity of these transcription factors on their specific target gene [13]. For example, BRCA1 promoter, which is lacking consensus EREs [14], is activated by the E2-liganded ERα. It has been noted that this activation is induced by a non-classical pathway, in which the E2-liganded-ERα binds to the p300 co-activator. Then, this E2-ERα-p300 complex, interacts with the Jun/Fos transcription factor which is linked to its DNA specific AP-1 site residing in the BRCA1 promoter [13]. Other studies have shown that this E2-induced BRCA1 expression requires recruitment of additional co-factors, such as the specific protein 1 (Sp1), the cyclic AMP responsive element binding (CREB) protein [15], the non-liganded aromatic hydrocarbon receptor (AhR) [16], the E2F transcription factor family [17], the member of the steroid receptor co-activators 1 and 3 [SRC1 [15] and SRC3 [18]] and certain other non-classical co-factors [12].

The human T-cell leukemia virus type 1 (HTLV-1) has been firmly implicated with the etiology of the aggressive malignancy adult T-cell leukemia (ATL) [19] and the neurological progressive inflammatory syndrome, called tropical spastic paraparesis or HTLV-1 associated myelopathy (TSP/HAM) [20]. In addition, there are few reports on HTLV-1 implication with several other clinical disorders [21]. The pathogenic mechanism of HTLV-1 has not been fully resolved yet [22]. Accumulating studies suggest that the HTLV-I basic leucine zipper factor protein (HBZ), originally discovered by Gaundray et al. [23], plays a major role in the ATL pathology [24], while other reports attribute such a role to the HTLV-1-mediated modulation of cellular miRNAs expression [25]. However, the viral Tax oncoprotein is most widely regarded as the crucial factor for initiating the leukemic process of ATL [26] and the neuro-inflammatory steps of TSP/HAM [20] and of certain other HTLV-1-related diseases, whereas the subsequent progression of these diseases are considered to be mediated by other specific factors [27].

Like BRCA1, Tax is also a multifunctional protein that interacts with multiple regulatory proteins and modulate their expression or functional activities, but while BRCA1acts as a tumor suppressor [8], [10], Tax is a potent oncoprotein [19]. Moreover, analysis of their biological effects and individual activities reveals that many of them are practically contrasting each other. For example; BRCA1 enhances DNA repair and thereby, reduces accumulation of cells with potentially tumorigenic mutations, whereas, Tax interferes with most of the DNA repair pathways and increases, thereby, the cell sensitivity to carcinogenesis [28]. In addition, BRCA1 provokes stress-induced cell-cycle arrest and apoptosis in cells remaining with DNA injuries due to escaping the repair process, which avoids their potential progress towards carcinogenesis [10]. However, Tax rather inhibits cell-cycle arrest and apoptosis in cells carrying DNA damage and enables their progression towards cancer. Of note, Tax can physically interact with a wide range of regulatory factors and modulate their transcriptional functions without its direct binding to the DNA. This includes interactions with some of the above-mentioned transcription activators and co-activators, which associate with ERα to mediate the E2-activation of BRCA1 expression. For example, Tax can bind the CBP/p300 and p/CAF co-activators/co-factors [29], recruits them for enhancing, in non-classical manner, the transcriptional activity of selected DNA-bound transcription factors which it can physically bind with [30]. On the other hand, Tax can, in contrast, compete for these co-activators and suppress, thereby, the expression of other genes that require CBP/p300-p/CAF factors for their transcription but cannot physically bind to Tax [31]. Furthermore, Tax can also bind other co-activators or co-factors like SRC-1 [32], E2F factor [33] and AhR [34], which, as noted above, are all involved in E2-induced BRCA1 activation and use them for competitive inhibition of BRCA1 activation.

In view of this established data, it was reasonable to postulate that if Tax and BRCA1 were acting within the same host cells, the potent Tax tumorigenic activities would likely antagonize BRCA1expression and its various tumor suppressing actions. This presumption prompted us to assess these putative Tax effects on BRCA1 in breast cells. We decided, here, to check, first, the influence of Tax on the E2-induced BRCA1 expression by testing the effect of Tax-expressing vector (CMV-Tax) on the expression of a Luciferase reporter driven by the BRCA1 promoter (BRCA1-Luc) in cultured breast cell lines. These experiments have revealed, for the first time, that Tax can block the E2-induced BRCA1 activation, which is mediated by the ERα-recruited p300/CBP cofactors in cancerous and non-cancerous cell-lines. We have demonstrated that Tax exerts this effect by blocking the ERα-non-classical AP1-associated transcriptional pathway and partially revealed the molecular mechanism of this effect.

## Materials and Methods

### Cells and Culture Conditions

In this study we used the weakly invasive ER-α positive MCF-7 epithelial-like cells and the highly invasive ER-α negative MDA-MB-231fibroblast-like breast cancer cells [35], obtained from Etta Livne, and the non-tumorigenic immortalized breast epithelial MCF-10A cells [36], given by Yacob Weinstein, both from our department. The Jurkat T-cell line was provided by Irvin Chen (Center for HIV and Digestive Diseases, UCLA, USA).

The MCF-7 and MDA-MB-231 cells were maintained in Dulbeco’s Modified Eagle’s Medium (DMEM) supplemented with 2 mM L-glutamine and 10% fetal bovine serum (FBS). The MCF-10A cells were cultured in DMEM/F12 (Invitrogen) containing 5% horse serum (Invitrogene), 0.5 mg/ml hydrocortisone (Sigma-Aldrich), 20 ng/ml epidermal growth factor (Sigma-Aldrich), 100 ng/ml cholera toxin (Sigma-Aldrich), 10 mg/ml insulin (Sigma-Aldrich). Jurkat T-cells were maintained in RPM11640 medium with 10% FBS. All above media were supplemented with 1% penicillin/streptomycin.

### Plasmids and Transfection

The reporter firefly luciferase (Luc) driven by the BRCA1 promoter (BRCA1-Luc) and Luc reporter driven by HTLV-1 LTR (LTR-Luc) were provided by Haim Werner (Clinical Biochemistry, Tel-Aviv University, Israel) and Susan J. Marriott (Baylor College, Houston, TX). The CBP and p300 plasmids were provided by addgene company. The ERα-expressing pCDNA3 vector [37] was from Michael Danilenko (Clinical Biochemistry, Ben-Gurion University, Beer-Sheva, Israel). The plasmid expressing 53PB1 was obtained from Prof. Michal Goldberg (Natural Sciences, Hebrew University, Jerusalem). The plasmid expressing the Renilla luciferase, was purchased from Promega (Madison WI, USA) [38] and Luc reporter driven by a minimal promoter linked to 3 copies of the consensus NF-κB responsive element (NF-κB-Luc) [38] was purchased from Clontech Laboratories (Palo Alto, CA). Francoise Bex (Universite Libre de Bruxelles, Belgium) provided the plasmids expressing the following CMV promoter-driven Tax mutants [39]: a) TaxM22 carrying the T130A, L131S dual nucleotide substitutions, capable of binding CREB, CBP/p300 and the p300/CBP-associated factor (p/CAF), but unable to dissociate the NF-κB factors from their IκB inhibitors, b) TaxM47 carrying the substitution L319R, L320S, capable of dissociating the NF-κB factors from their IκBs and binding CREB and CBP/p300, but incapable of binding p/CAF, which was essential for activating CREB pathway, thus it could not activate CREB pathway [40]. TaxV89A mutant obtained from Chou-Zen Giam (Uniformed Service University, Bethesda, USA), This mutant could bind p/CAF and activate the NF-κB factors, but could not bind the CBP/p300 and therefore, it was unable to activate the CREB- nor the NF-κB-associated pathways by itself, but when it was co-expressed with TaxM22 or TaxM47 it could complement their defects [39].

The plasmids were transfected at the indicated combinations by jetPRIMTM kit (Polyplus, www.polyplus-transfection.com) according to the manufacturer’s instructions. The transfection efficiency, determined with GFP-expressing plasmid, was found by FACS analysis to range in our cells between 70 to 80% (not shown). Each transfection mixture included the pRL-renilla plasmid (0.2 µg) as control for transfection efficiency. The cells were harvested at 24 hr post-transfection for measuring the enzymatic activities. Where specified, 20 nM Estrogen (E2, Sigma-Aldrich Chemical Co.) was added to the cultures 5 hr before cell harvest. The Luc activity was normalized to that of renilla and presented as fold of the relevant control.

### Measurement of BRCA1 mRNA Levels

Total cellular RNA was extracted from the tested cells using RNA extraction kit (Invitrogen). Total RNA was then reverse transcribed for cDNA generation using random primers and Moloney murine leukemia virus reverse transcriptase (both from Invitrogen) as described by the manufacturer. A quantitative real time PCR method was used to measure BRCA1 mRNA expression as previously described [41] using the AB 7300 real-time PCR system. Primer sequences for BRCA1 were forward 5′-GGCTATCCTCTCAGAGTGACATTT-3′ and reverse 5′-GCTTTATCAGGTT ATGTTGCATGGT-3′, as previously described (19). The PCR product size generated with these primers was 69 bp. The primers sequences for the endogenous reference gene, β-actin, were: forward 5′-TGA GCG CGG CTA CAG CTT-3′, reverse 5′-TCC TTA ATG TCA CGC ACG ATT T-3′. Relative BRCA1 mRNA expression was quantified using the standard curve method. For accurate normalization purposes, the linearity of the PCR amplification reactions for endogenous reference genes was confirmed as comparable to BRCA1 primers (data not shown). Results obtained were the mean of two independent experiments.

### Antibodies, Cell Fractionation, Co-immunoprecipitation and Western Blot Analyses

Monoclonal antibodies against BRCA1, Tax, CBP and p300 were all purchased from Santa Cruz Biotechnology Inc (Santa Cruz, CA, USA).

Whole cell extracts and sub-cellular fractions were prepared by NucBuster Kit (Calbiochem, Catalog No. 71183–3) according to the Manufacturer’s protocol.

For co-immunoprecipitation assays, aliquots of the nuclear extracts (200 µg protein) were immunoprecipitated with the specified mouse antibodies and analyzed by Western blot for co-precipitated proteins with the respective rabbit antibodies as previously described [42].

For direct Western analyses, aliquots of the tested extracts (80 µg protein) were analyzed with the respective antibodies as described elsewhere [43].

### Chromatin Immunoprecipitation (ChIP)

MCF-7 (2×10^7^) cells were transfected with Tax expressing plasmid jetPRIMTM kit and at 24 hr post-transfection the cells were treated with E2 for 5 hr. The chromatin mmunoprecipitation was performed by EZ Chip kit (Millipore) according to the manufacturer’s instruction. The BRCA1 promoter region flanking the Sp/AP-1/CRE binding sites (171 base pairs) in the obtained DNA was amplified by real time PCR, using the following primers: forward:

5′-GACAGATGGGTATTCTTTGACG-3′ and reverse: 5′-GCATATTCCAGTTCC TATCACGAG-3′ [15].

## Results

### Assessing the Expression and Functionality of the Employed Ectopic Tax Variants in Cancerous and Non-cancerous Human Breast Epithelial Cell Lines

**Figure 1:**
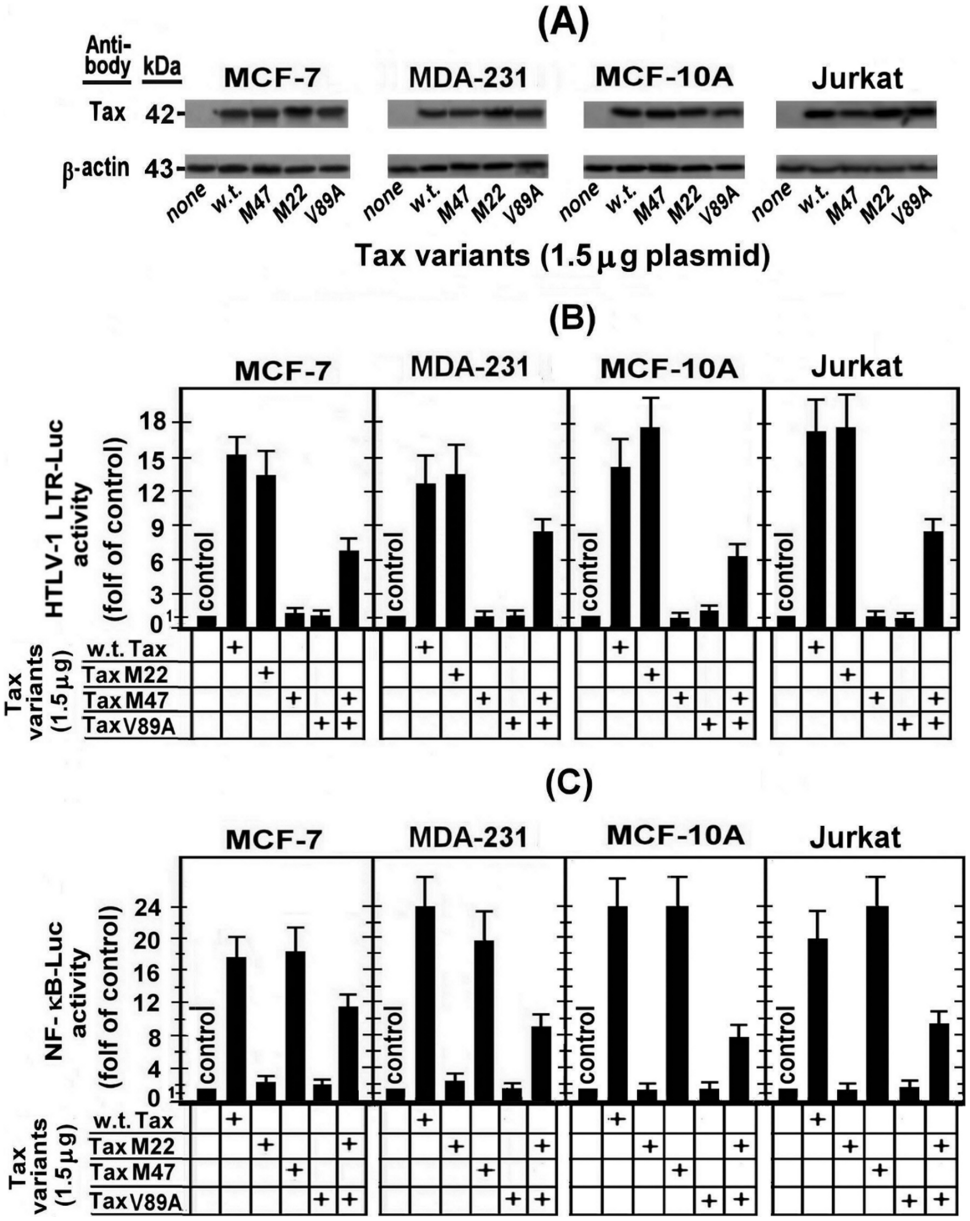
Assessing the expression and functionality of the employed ectopic Tax variants in cancerous and non-cancerous human breast epithelial cell lines. (A) The tested cells were_transfected with equal doses (1.5 µg) of the Tax varients plasmids and their Tax protein levels were determined at 24 hr post-transfection by Western blot analysis of the whole cell extracts with Tax monoclonal antibody. Equal sample loading was assessed by re-processing the blot with anti actin antibody. The effect of Tax varients on HTLV-1 LTR-Luc reporter (B) and on the NF-κB-Luc reporter (1C) was examined by co-transfecting the appropriate cells with 1.5 µg of either of these repoters and 1.5 µg of each of the tested Tax varients. Luciferase activity was measured in the cell lysates at 24 h post-transfection. The presented results are an average of three repeated experiments ± SE.

The present study was undertaken to investigate the influence of HTLV-1 Tax on the E2-induced BRCA1-Luc expression in various breast cell lines. This was done by testing the effect of ectopic Tax-expressing vector (CMV-Tax) on RBCA1-Luc in the cancerous MCF-7 and MAD-MB-231 and the non-cancerous MCF-10A breast cells. Since Tax is known to modulate the expression and function of many target genes via CREB or NF-κB-associated pathways [28], [30] and by recruitment of transcriptional co-activator/co-factors, we initiated this study by exploring whether Tax exerts its effects on BRCA1 expression through any of these three pathways. For this purpose, we employed the wild type (w.t.)Tax and its three mutants TaxM22, TaxM47 and TaxV89A which are described in “Materials and Methods”. For control of Tax activity in human non-mammary cells, we used the human Jurkat T-cells in order to verify that our results are not influenced by cell-type specific side effects. The expression of these Tax variants was determined by transfecting the cells with equal doses (1.5 µg) of their plasmids and measuring their protein level at 24 hr post-transfection by Western blot analysis of the whole cell extracts with Tax monoclonal antibody. Figure 1A demonstrates that the expression of all the Tax variants in the breast cells was similarly high as in the control Jurkat cells.

Next we elucidated whether Tax variants could retain their genetic functionality during their expression in the tested cells. This was done by testing their effect on HTLV-1 LTR-Luc (Figure 1B), which required the CREB-pathway for its activation, and the NF-κB-Luc reporter (Figure 1C) whose activation is dependent on the NF-κB-pathway. These experiments revealed that w.t.Tax activated both reporters. However, TaxM22 activated only LTR-Luc, TaxM47 could activate only the NF-κB-Luc and TaxV89A could not induce any of these reporters. In addition, when TaxV89A was co-expressed with TaxM47 (Figure 1B) or TaxM22 (Figure 1C) it could complement their defects [39]. Taken together, the observations depicted in Figures 1A, 1B and 1C confirmed that the expression of these different Tax variants and their activation pathways were not cell-type specific.

### Effect of Tax on BRCA1 Activation by E2 and 53PB1

**Figure 2:**
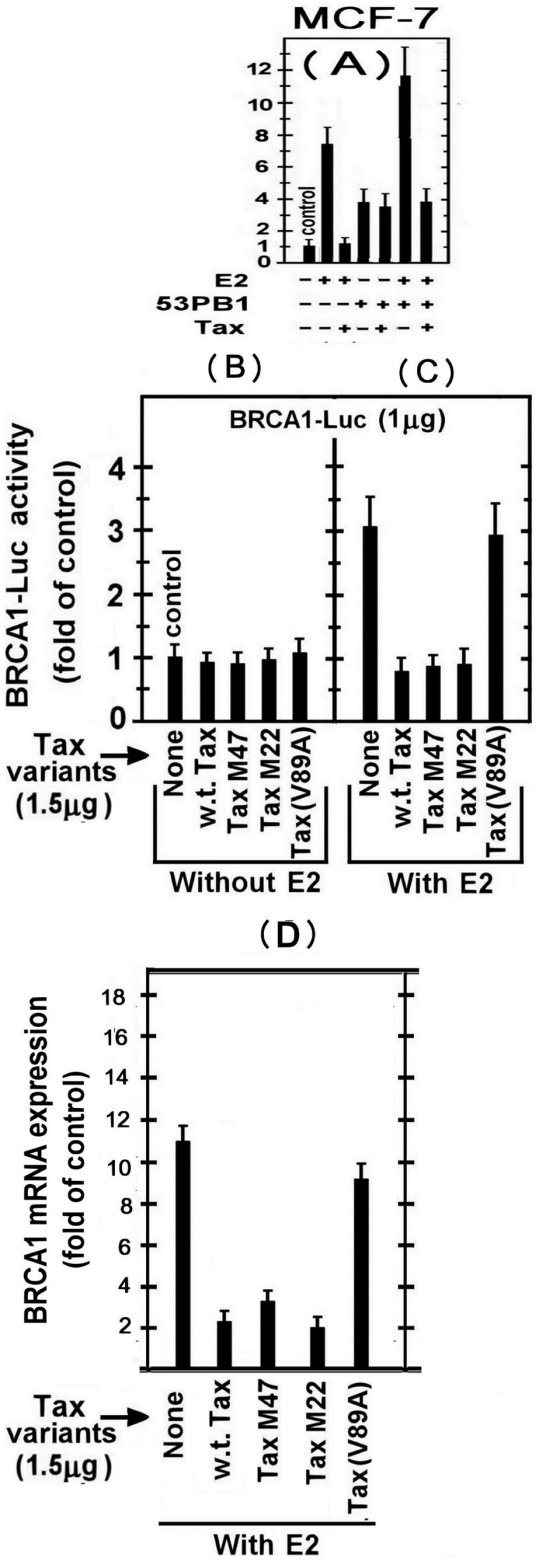
Effect of Tax on BRCA1 activation by E2 and 53PB1. (A) MCF-7 cells were co-transfected with 1.5 µg of BARCA1-Luc reporter and the plasmids expressing 53PB1 and/or Tax. Where indicated, E2 (20 nM) was added to the cultures 5 hr before harvesting the cells for analyzing the reporter expression. (B) and (C) MCF-7 cells were co-transfected with 1.5 µg of BARCA1-Luc reporter and the plasmids expressing different Tax varients without (B) or with (C) E2 treatment. As above, E2 was added 5h before harvesting the cells for Luciferase activity measurement in the cell lysates at 24 h post-transfection. (D) MCF-7 cells were co-transfected with 1.5 µg of the plasmids expressing different Tax variants with E2 treatment and at 24h post transfection the BRCA1 mRNA levels were examined as detailed in “Material and Methods” section. The presented results are an average of three repeated experiments ± SE.

In the experiment presented in Figure 2A we examined the effect of w.t.Tax on BRCA1-Luc expression in MCF-7 cells, which were chosen as representative breast cells. The results of this experiment show that E2 activated BRCA1 expression by 7–8 folds of its basal level and that Tax drastically inhibited this activation. To explore whether Tax is a general inhibitor of BRCA1 activation or is rather specific to selective BRCA1 activators, we arbitrarily tested its effect against 53PB1, which has been formerly reported to activate BRCA1 in absence of E2 [44]. Figure 2A shows that in E2 absence 53BP1 increased BRCA1 expression to a 4 fold higher than its basal expression, whereas Tax had no significant effect on this stimulation. Furthermore, this experiment revealed that when E2 and 53BP1 were applied together, they exerted an additive stimulation, indicating that their stimulatory effects on BRCA1 expression were unaffected by each other. This independency between E2 and 53PB1was further substantiated by showing that Tax lowered the combined stimulatory effect of E2+53PB1 to the level obtained by 53BP1 alone, which re-confirmed that E2- but not 53PB1-mediated stimulation was susceptible to Tax inhibition. The mechanism of 53PB1 stimulatory effect on BRCA1 and its interaction with Tax is currently under a separate investigation.

### BRCA1 Expression is Inhibited by w.t.Tax and its M47 and M22 Mutants but not by TaxV89A

As noted before, Tax can modulate the expression or functions of many gene products through CREB and NF-κB-associated pathways or by recruitment of the CBP/p300 co-factors. To obtain an initial clue on the mode of Tax-induced inhibition of E2-stimulated BRCA1 expression, we elucidated whether any of above major pathways might be involved in this Tax activity. In addressing this question, we found that none of the Tax variants affected the basal level of BRCA1 expression (Figure 2B). However, the w.t.Tax, as well as TaxM47 and TaxM22 mutants could inhibit E2-mediated BRCA1 expression (Figure 2C), thus excluding the involvement of both CREB and NF-κB in this inhibition of BRCA1 expression. Notably, however, TaxV89A, which could not bind the CBP/p300 co-factors [39], failed to inhibit this E2-mediated BRCA1 expression (Figure 2C). Together, these findings suggest that the inhibition of the E2-induced BRCA1 expression by Tax requires its interaction with CBP/p300.

In order to examine possible effect of Tax on BRCA1 transcription, BRCA1 mRNA levels were examined in these cells transfected with Tax variants. In agreement with the above results, it can be seen that w.t.Tax, as well as TaxM47 and TaxM22 mutants strongly inhibited BRCA1 mRNA expression, whereas TaxV89A had no effect on this expression (Figure 2D).

### Tax Inhibits the Non-classical Pathway of E2-ERα Induced BRCA1 Expression

**Figure 3:**
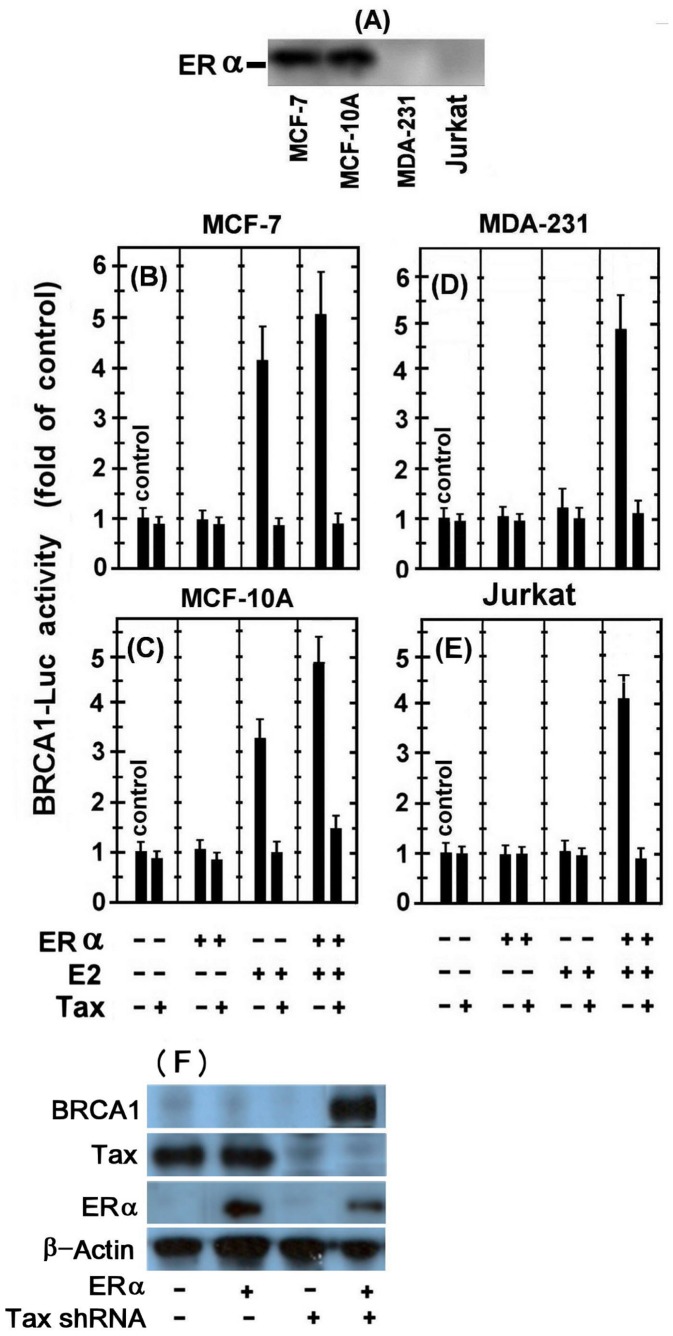
Effect of Tax on the non-classical pathway of E2-ERα induced BRCA1 expression. Western blot analysis of the whole cell extracts of the examined cell lines with anti ERα monoclonal antibody. MCF-7 (B), MCF-10A (C), MDA-231 (D) and Jurkat (E) cells were transfected with either the BRCA1-Luc (1.5 µg) alone or together with 1.5 µg of the indicated combinations of the ERα or the w.t.Tax expressing plasmids. Where indicated, E2 was added to the cultures 5 hr before harvesting the cells for analyzing the reporter expression. The presented results are an average of three repeated experiments ± SE.

Next, it was important to explore the effect of Tax on BRCA1 expression in ERα containing versus lacking breast cells. However, due to discrepant reports in the literature regarding the presence of ERα in MDA-123 and MCF-10A cells, we re-assessed first, this issue in our employed cell lines by Western blot analysis of their whole cell. Figure 3A shows that while ERα was readily detected in MCF-7 and MCF-10A, it was not found in the MDA-123, nor in the Jurkat control cells.

The effect of Tax on BRCA1 expression in these different cells was examined by transfecting the cells with either the BRCA1-Luc alone or together with the indicated combinations of ERα or w.t.Tax expressing plasmids. Where indicated, E2 was added to the cultures 5 hr before harvesting the cells for analyzing the reporter expression. This experiment revealed that introducing the ectopic ERα-expressing plasmid without E2-treatment, had no significant effect on the reporter expression in any of the employed cells (Figures 3B to 3E). On the other hand, E2 treatment without introducing the ectopic ERα plasmid markedly enhanced the reporter expression in the MCF-7 (Figure 3B) and MCF-10A (Figure 3C) but not in MDA-231 (Figure 3D) and Jurkat (Figure 3E) cells. Furthermore, while the ectopic ERα plasmid substantially stimulated the reporter expression in the E2-treated MDA-231 (Figure 3D) and Jurkat (Figure 3E) cells (by 4–5 fold), it only moderately (25–50%.) increased the E2-stimulated reporter expression in MCF-7 (Figure 3B) and MCF-10A (Figure 3C) cells. These data are consistent with the well-established concept that E2 stimulates BRCA1 expression by liganding to ERα and activating, thereby its transcriptional function. Notably, Tax was found to strongly inhibit the stimulated reporter expression in all of these experimental settings.

In order to examine the effect of Tax in its physiological conditions on ERα induced BRCA1 expression, MT2 cells (infected T-cells with HTLV-1) were transfected with ERα alone or together with Tax shRNA expressing plasmids and treated with E2. BRCA1 and Tax protein levels were measured at 24 hr post-transfection by Western blot analysis of the whole cell extracts with the corresponding monoclonal antibodies. Fig. 3F shows that introducing ERα into these cells, which expressing HTLV-1 Tax protein, could not significantly elevate BRCA1 protein expression, while when the synthesis of Tax protein was silenced by its specific shRNAs, high levels of BRCA1 protein expression were detected.

### Effect of CBP/p300 on Tax Inhibition of the E2-stimulated BRCA1 Expression

**Figure 4:**
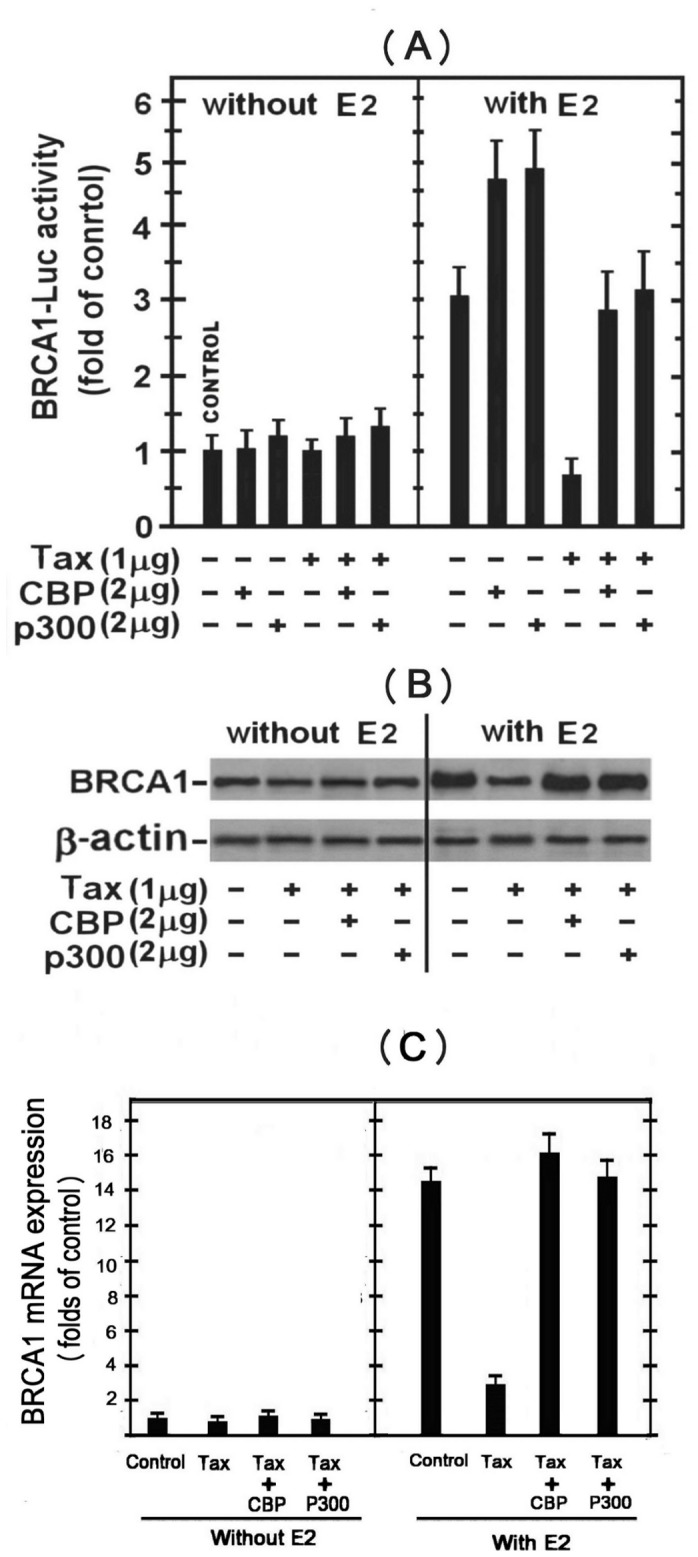
Effect of CBP/p300 on Tax inhibition of the E2-stimulated BRCA1 expression. (A) MCF-7 cells were transfected with either the BRCA1-Luc (1.5 µg) alone or together with the indicated combinations of w.t.Tax, CBP or p300 expressing plasmids without (left lane) or with (right lane) E2 treatment. The E2 was added to the cultures 5 hr before harvesting the cells for analyzing the reporter expression. (B) BRCA1 protein levels in the different transfected MCF-7 cells detailed in (A) were detected by Western blot analysis of the whole cell extracts with anti BRCA1 antibody. Equal sample loading was assessed by re-processing the blot with anti actin antibody.

To assess further the possibility that Tax acts by competing for CBP/p300 as described in Figure 2, we examined the effect of elevating the cellular level of these co-activators by their ectopic expression in MCF-7 cells. The left panel of Figure 4A shows that Tax has no significant effect on the basal expression of the BRCA1 reporter in the E2-non-treated MCF-7 cells. Introducing ectopic CBP or p300 had no effect on the basal expression of this reporter. In contrast, the right panel of this Figure shows that each of these ectopic co-activators further enhanced the E2-stimulated expression of the BRCA1 reporter and almost completely alleviated the Tax-induced strong inhibition of the E2-stimulated reporter expression.

Figure 4B shows that the effects of Tax, E2 and CBP/p300 on BRCA1 transcriptional expression were reflected also in its protein level detected by Western blot analysis of the whole cell extracts with anti BRCA1 antibody. In E2-non-treated cells, neither Tax nor CBP or p300 had significant effect on the basal level of BRCA1 protein (left panel). Conversely, E2 treatment markedly elevated the BRCA1 protein level, which was strongly suppressed by Tax and fully restored by the ectopic co-activators (right panel). Collectively, the results depicted in Figure 4A and 4B suggest that Tax inhibits the E2-stimulation of the BRCA1 expression by a mechanism involving the CBP/p300 co-activators.

### Tax Physically Associates with the ERα-CBP/p300 Complex through Binding to the Recruited CBP/p300

**Figure 5:**
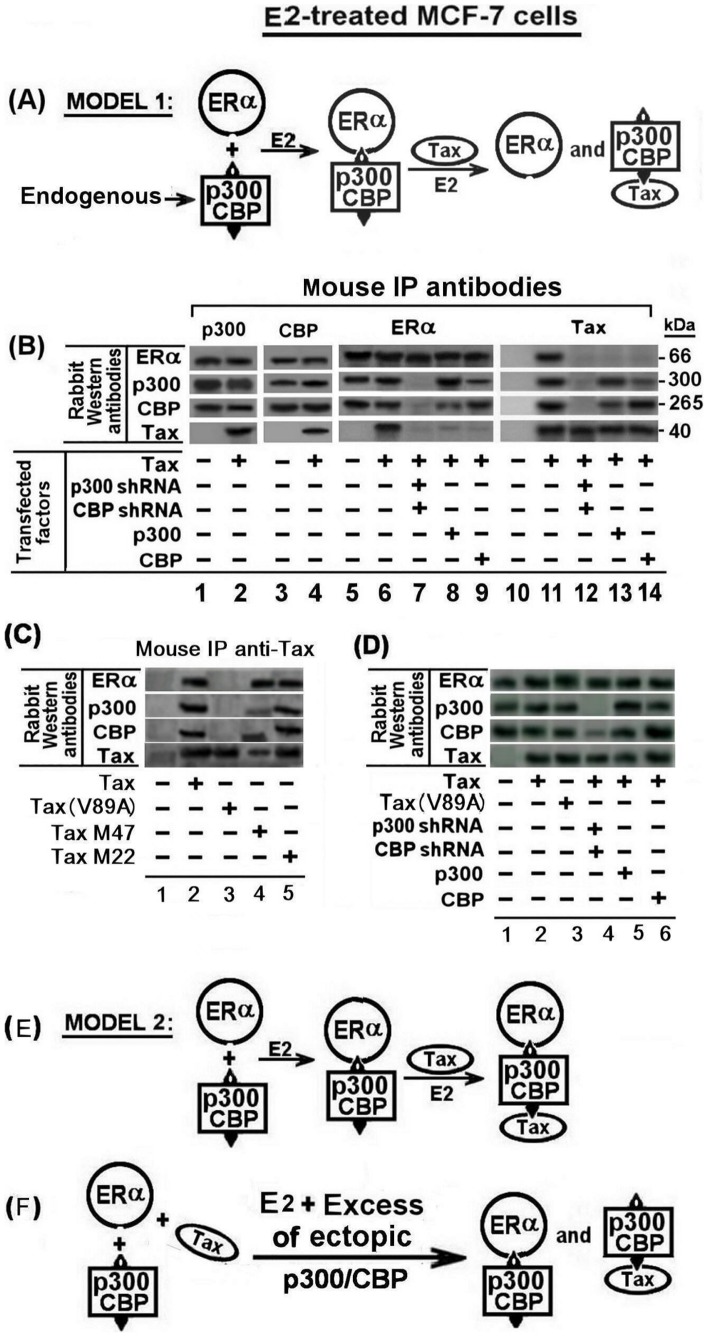
Tax physically associates with the ERα-CBP/p300 complex through binding to the recruited CBP/p300. (A) Schematical model 1 describing the formation of separate ERα-p300/CBP and Tax- p300/CBP complexes in E2 treated breast cells with or without Tax expression. (B) MCF-7 cells were transfected with 1.5 µg of the indicated combinations of w.t.Tax, p300 shRNA, CBP shRNA, p300 and CBP expressing plasmids. The cells were treated with E2 at 5 hr before extracting the cells for coimmunoprecipitation (co-IP) analyses. The whole cell extracts were immunoprecipitated with p300, CBP, ERα and Tax mouse specific monoclonal antibodies as indicated in the figure. The various immunoprecipites were analyzed by Western blot analysis with ERα, p300, CBP and Tax rabbit specific monoclonal antibodies. (C) MCF-7 cells were transfected with 1.5 µg of w.t.Tax or each of its variants V89A, M22 and M47 expressing plasmids. The cells were treated with E2 at 5 hr before extracting the cells for coimmunoprecipitation analyses. The whole cell extracts were immunoprecipitated with Tax mouse specific monoclonal antibody. The various immunoprecipites were analyzed by Western blot analysis with ERα, p300, CBP and Tax rabbit specific monoclonal antibodies. (D) Western blot analysis of the protein expression of ERα, p300, CBP and Tax in the lysates of the cells extracts of all the different transfections in part (B) before co-IP. (E) Schematical model 2 describing the formation of the ERα-p300/CBP-Tax tertiary complex complexe in E2 treated breast cells with Tax expression. (F) Schematical model describing the formation of separate ERα-CBP/p300 and Tax-CBP/p300 complexes_in E2 treated breast cells with Tax and excessive level of CBP/p300 expression.

As noted above, Tax has been reported to suppress CBP/p300 dependent expression of certain genes by sequestering these co-activators [31]. Furthermore, Jeffy et al [13] have reported that p53, which requires CBP/p300 for its transcriptional function, inhibits the ERα-mediated stimulation of BRCA1 expression by E2. Based on this information it could be assumed that Tax inhibited the E2-induced BRCA1 stimulation by competing for p300/CBP and avoiding, thereby, the ERα-p300/CBP complex formation as schematically illustrated in Figure 5A. However, this presumption was refuted by our coimmunoprecipitation analyses of the E2-treated MCF-7 cell extracts presented in Figure 5B. These analyses revealed that the immunoprecipitates pulled by mouse p300 (lanes 1 and 2) or CBP (lanes 3 and 4) specific antibody included practically the same amounts of ERα protein regardless of whether or not the cell were transfected with ectopic Tax (compare lane 1 with 2 and lane 3 with 4). In addition, each of the precipitates obtained by the antibodies of these co-activators included also appreciable amounts of their reciprocal co-activators (see the CBP bands in the precipitates obtained by the anti p300 antibody and the p300 bands in the precipitates obtained by the anti CBP antibody). These bands reflected the physical linkage of the reciprocal co-activators with the co-precipitated ERα protein. However, quite surprisingly, Tax was also co-precipitated by these two antibodies (see lane 2 and 4 for the p300 and CBP antibodies respectively). This finding suggests that Tax did not prevent the binding of CBP/p300 to ERα but rather physically associated with the ERα-CBP/p300 complex to form an ERα-CBP/p300-Tax complex. This presumption was further supported by our next observation that the precipitate pulled down from the Tax-transfected cells by the ERα specific antibody included the two co-activators, as well as the Tax protein (see lane 6) and conversely, the precipitate obtained from these same cells by the Tax specific antibody included the ERα protein. Notably, in this context, the CBP/p300 co-activators have been shown by Ramirez et al, and Scoggin et al. [29], [32] to include several domains on their proteins for physical association with Tax and certain other transcription-modulating factors. It is, therefore, more convincing to assume that Tax associates with the ERα-CBP/p300 complex through binding to the co-activators rather than through binding to the ERα protein which firstly presumed in the illustrated Model 2 presented in Figure 5E. This presumption was supported by our finding that when the synthesis of the CBP/p300 co-activators was silenced by their specific shRNAs, Tax was unable to bind directly to ERα (see lanes 7 and 12). Also, it was supported by our coimmunoprecipitation analyses of MCF-7 cells transfected with the different Tax variants and pulled by mouse Tax monoclonal antibody (Figure 5C). These analyses revealed that the immunoprecipitates of the cells transfected with w.t.Tax, TaxM22 or TaxM47 (see lanes 2, 4 and 5) included significant amounts of ERα, CBP and p300 proteins. However, the immunoprecipitates of the cells transfected with Tax(V89A), which could not bind the CBP/p300 co-factors, (see lane 3) did not include any of these proteins.

### Excessive Level of CBP/p300 Prevents Tax Binding to the ERα-CBP/p300 Complex

As shown in Figure 4A, increasing the level of the CBP/p300 co-activators by their ectopic overexpression abolished the Tax-inhibitory effect on the E2-stimulated BRCA1 expression.

To explore the molecular process of alleviating this Tax inhibitory effect, we examined the effect of ectopic overexpression of CBP or p300 on Tax interaction with the ERα-CBP/p300 complex by co-immunoprecipiton analyses. Figure 5B shows that the precipitate obtained by anti ERα antibody from the E2-treated cells with ectopic p300 overexpression, contained large amount of p300 and considerably smaller amount of CBP (lane 8). On the other hand, the precipitate obtained by anti ERα antibody from the E2-treated cells with ectopic CBP overexpression, contained large amount of CBP and smaller amount of p300 (lane 9). Notably, however, neither of these immunoprecipitates included the Tax protein (see the bottom of lanes 8 and 9). Furthermore, the immunoprecipitates pulled down from these cells by anti Tax antibodies, contained the same relative amounts of the p300 and CBP (lanes 13 and 14) as the former two precipitates which were obtained by the anti ERα antibody. However, neither of these latter precipitates contained the ERα protein (see the top of lanes 13 and 14). These findings are consistent with the schematic model illustrated in Figure 5F, which suggests that the excessive CBP/p300 enables ERα and Tax to separately form their own ERα-CBP/p300 and Tax-CBP/p300 complexes, while avoiding Tax from physical interaction with the BRCA1-activating ERα-CBP/p300-complex.

### Tax Prevents ERα-CBP/p300 Complex Binding to the BRCA1 Promoter

**Figure 6:**
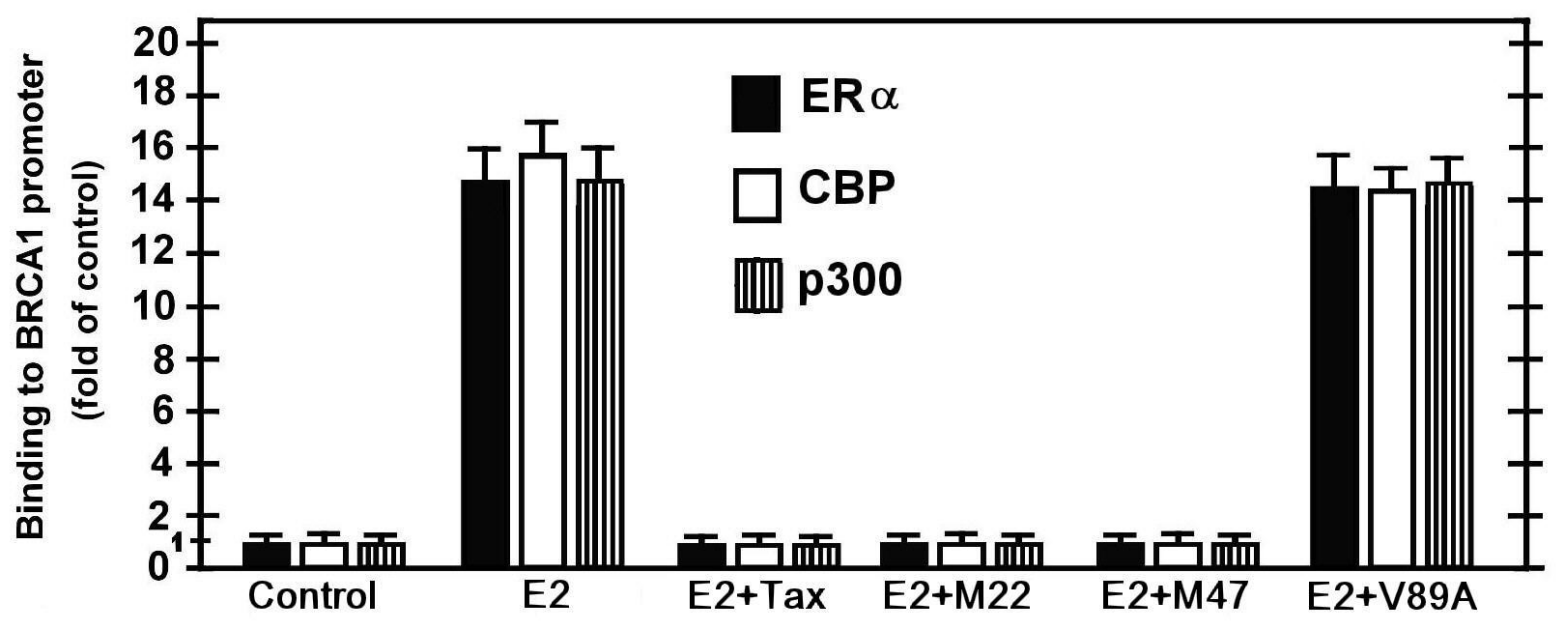
Effect of Tax on ERα-CBP/p300 complex binding to BRCA1 promoter. MCF-7 cells which were or not transfected with 1.5 µg of Tax variants [w.t.Tax, TaxM22, TaxM47 or Tax(V89A)] were treated with E2 at 5 hr before their extraction for examining the binding of ERα, CBP and p300 proteins to BRCA1 promoter by CHIP assay as described in Materials and Methods section. Control cells were not transfected with Tax and not treated with E2. The presented results are an average of three repeated experiments ± SE.

Earlier reports [13], [45] have shown that the E2-ERα- CBP/p300 complex binds to the BRCA1 promoter by linking to the Jun/Fos or Jun/Jun transcription factors residing at the AP1 site in the BRCA1 promoter. In view of our above findings that rull out the possibilty that Tax inhibitst the E2-ERα-induced BRCA1 activation by preventng the E2-ERα-CBP/p300 complex formation, we elucidated whether Tax prevented the final step of the E2-ERα-CBP/p300 complex binding to the AP-1 DNA site at the BRCA1 promoter. This was done by the ChIP analysis depicted in Figure 6, which confirmed that w.t.Tax and its variants (TaxM22 and TaxM47) blocked the E2-ERα-CBP/p300 complex binding to the AP-1 DNA site of the BRCA 1 promoter, whereas Tax (V89A) had no effect on the binding of this complex to BRCA 1 promoter.

## Discussion

The effect of HTLV-1 Tax oncoprotein on the expression of BRCA1 in E2-treated breast cells and its molecular mechanism were investigated in this study. Consistent with earlier reports [14], [15], [16], we demonstrated that E2 profoundly induced BRCA1 expression in various breast cell lines by liganding its ERα receptor. We also confirmed that this activation was excerted via the non-classical ERα pathway by showing that the E2-ERα complex indirectly attached to the BRCA1 DNA promoter by linking to Jun-Fos or Jun-Jun localizing on its AP1 site. In addition, consistent with early reports [45], we have noted that the E2-ERα complex recruits the CBP/p300 trascription co-factors for co-operative enhancement of BRCA1 promoter expression. Notably, however, we demonstrate here, for the first time a unique observation that the HTLV-1 Tax oncoprotein drastically antagonizes this E2-ERα mediated activation of BRCA1 (Figure 6). Previous studies have shown that Tax is capable of blocking the expression of certain genes by competing for their recruited transcriptional co-activators, such as CBP/p300, which are essential also for BRCA1 expression [31]. In line with such crossing reports, we observed that increasing the intracellular level of CBP/p300 co-factors by ectopic overexpression abolished the Tax-inhibitory effect of the E2-stimulated BRCA1 expression (Figure 4). Therefore, we examined whether Tax inhibited BRCA1 activation by competing for p300/CBP cofactors and avoiding, thereby, the ERα-p300/CBP complex formation. However, this presumption was ruled out by our finding which proved that Tax exerted this inhibition by binding to the CBP/p300 co-factors without separating them from ERα, but rather by forming an ERα-CBP/p300-Tax tertiary complex (Figure 5C). Furthermore, we proved that Tax could not contact directly with ERα molecule by showing that silencing the CBP/p300 co-factors synthesis with their specific shRNAs, avoided Tax binding to ERα (Figure 5). In line with this, the CBP/p300 factors have been proved to contain several domains for binding of Tax and certain other transcription factors [29], [32]. Moreover, our reciprocal co-immunoprecipitation analysis revealed that excessive intracellular CBP/p300 level enables ERα and Tax to separatedly form ERα-CBP/p300 and Tax-CBP/p300 complexes without competing with each other for the CBP/p300 co-activators and avoiding also Tax from physically interacting with the ERα-CBP/p300 complex (Figure 5). These findings, together with the Chip assay data (Figure 6), provided strong indications that Tax inhibits the E2- ERα-CBP/p300-mediated BRCA1-activation by blocking the access of the whole transcriptional complex to the AP1 site of the Jun-Jun/Jun-Fos at the BRCA1 promoter.

Based on these data it is tempting to speculate that penetration of HTLV-1 into breast epithelial cells would likely sensitize them to high risk for malignant transformation by environmental and other source of carcinogens. Interesting studies related to this context, have been reported with HTLV-1 infected women in endemic area. Such women are strongly advised to avoid or to limit long-term breast-feeding in order to avoid, or minimize, the virus transition to their infants. Milk of HTLV-1 infected lactating women is highly loaded with HTLV-1 producing T-lymphocytes as well as with large numbers of already infected breast epithelial cells [46], [47]. Southern et al. [46] have shown that breast-milk epithelial cells and other epithelial cells can be infected with HTLV-1 by co-cultivating them with breast milk-born HTLV-1 producing T-cells and these infected epithelial cells can, in turn, infect other epithelial and T-cells. Furthermore, these authors have speculated that such infected epithelial breast cells might be the reservoir source for the high rate of the beast-feeding route of the HTLV-1 transmission from mothers to infants in endemic areas. Also, this ability of HTLV-1 infection of different epithelial cells was proved by other different previous studies [48], [49], [50], [51]. Unfortunately, only two poorly designed surveys have been reported so far in the literature that has come up with unconvincing negative data. In the first publication [52] the authors examined the presence of HTLV-1 in patients with different kinds of cancers, including breast cancer. Although they did not find higher rate of HTLV-1 infected persons among the breast cancer patients, this approach was inadequate, because the general incidence of breast cancer in regular women population is estimated to be 8–12% whereas the incidence of HTLV-1 infected women even in highly enedemic areas is 1–5%. Therefore, the approach should be just the opposite, i.e. to estimate the incidence of breast cancer among HTLV-1 infected population of old women with habits of prolonged breast-feeding. In the second publication [53] the authors examined the development of different kinds of cancers, including breast cancer, in HTLV-1 infected Japanese population. However, these authors did not focus on old women with habits of prolonged breast-feeding. It should be emphasized that intensive and well-designed epidemiological studies for examining the involvement of HTLV-1 in breast cancer are still required, focusing on assessing whether the incidence of breast cancer might be significantly higher in chohorts of large numbers of elder HTLV-1 infected women in endmic areas especially in communities with traditionally prolonged breastfeeding [46].

We hope that our present and subsequent molecular studies, to be published soon from our laboratory, which will provide additional data on Tax and other HTLV-1 components and mechanisms, will attract more scientists to deeply elucidate further this issue in order to resorve it.

Although HTLV-1 Tax strongly inhibited BRCA1 gene expression in breast cells (through the non-classical pathway) as mentioned above, our un-shown data (to be reported elsewhere) proved a potent stimulatory effect of HTLV-1 Tax on E2-ERα transcriptional activity through the classical pathway. This pathway starts by a direct binding of the activated ERα to the EREs residing on the target promoters and ends by stimulating the transcription of the respective genes [12]. Also, it is known that part of these stimulated genes led to enhanced replication of the cells [54]. Taking together all these data, it is clear that, in one hand, HTLV-1 Tax accelerates cell replication through ERα classical pathway and probably other pathways such as NFkB [55], and on the other hand prevents the expression and possibly the functions of BRCA1. These combined activities in breast cells very likely can lead to breast cancer development.
